# A Simple Admission Order-set Improves Adherence to Canadian Guidelines for Hospitalized Patients With Severe Ulcerative Colitis

**DOI:** 10.1093/jcag/gwac032

**Published:** 2023-02-04

**Authors:** Steven Li Fraine, Isabelle Malhamé, Teresa Cafaro, Camille Simard, Elizabeth MacNamara, Myriam Martel, Alan Barkun, Jonathan M Wyse

**Affiliations:** Division of Gastroenterology, McGill University Health Centre, Montreal, Quebec, Canada; Division of Gastroenterology, Jewish General Hospital, McGill University, Montreal, Quebec, Canada; Division of General Internal Medicine, McGill University Health Centre, Montreal, Quebec, Canada; Division of General Internal Medicine, Jewish General Hospital, McGill University, Montreal, Quebec, Canada; Division of General Internal Medicine, Jewish General Hospital, McGill University, Montreal, Quebec, Canada; Department of Medical Biochemistry, Jewish General Hospital, McGill University, Montreal, Quebec, Canada; Division of Gastroenterology, McGill University Health Centre, Montreal, Quebec, Canada; Division of Gastroenterology, McGill University Health Centre, Montreal, Quebec, Canada; Division of Gastroenterology, Jewish General Hospital, McGill University, Montreal, Quebec, Canada

## Abstract

**Background:**

Individuals hospitalized with severe ulcerative colitis represent a complex group of patients. Variation exists in the quality of care of admitted patients with inflammatory bowel disease. We hypothesized that implementation of a standardized admission order set could result in improved adherence to current best practice guidelines (Toronto Consensus Statements) for the management of this patient population.

**Methods:**

A retrospective cohort study of patients admitted with severe ulcerative colitis to a Montreal tertiary center was conducted. Two cohorts were defined based on pre- and post-implementation of a standardized order set. Adherence to 11 quality indicators was assessed before and after implementation of the intervention. These included: *Clostridioides difficile* and stool cultures testing, ordering an abdominal X-ray and CRP, organizing a flexible sigmoidoscopy, documenting latent tuberculosis, initiating thromboprophylaxis, use of intravenous steroids, prescribing infliximab if refractory to steroids, limiting narcotics, and surgical consultation if refractory to medical therapy.

**Results:**

Adherence to 6 of the 11 quality indicators was improved in the post-intervention cohort. Significant increases were noted in adherence to *C difficile* testing (75.5% versus 91.9%, *P* < 0.05), CRP testing (71.4% versus 94.6%, *P* < 0.01), testing for latent tuberculosis (38.1% versus 84.6%, *P* < 0.01), thromboprophylaxis (28.6% versus 94.6%, *P* < 0.01), adequate corticosteroids prescription (72.9% versus 94.6%, *P* < 0.01), and limitation of narcotics prescribed (68.8% versus 38.9%, *P* < 0.01).

**Conclusions:**

Implementation of a standardized order set, focused on pre-defined quality indicators for hospitalized patients with severe UC, was associated with meaningful improvements to most quality indicators defined by the Toronto Consensus Statements.

## BACKGROUND

Individuals hospitalized with severe ulcerative colitis (UC) represent a complex group of patients. Since they are often cared for by clinicians from multiple disciplines and specialties including nursing, emergency medicine, family medicine, general internal medicine, and gastroenterology, a coordinated approach to care is paramount to effective and safe inpatient management. Canadian Guidelines titled ‘Treatment of hospitalized adult patients with severe Ulcerative Colitis - Toronto Consensus Statements’ address the inpatient clinical management of patients with severe UC flare (1). Despite these available guidelines describing a standardized approach to this patient population, variation exists in the quality of care and prevention of complications of admitted patients with inflammatory bowel disease (IBD) (2). For example, in a recent multicenter Canadian study of patients admitted for IBD-related problems, there were significant variations in inpatient quality indicators such as venous thromboembolism prophylaxis (VTE) and *Clostridioides difficile* testing across centers and admitting services (3). Jackson et al. showed that overall adherence to international guidelines for IBD management of admitted patients was 71%, whereas Law et al. revealed that variations exist even among gastroenterologist subspecialities (4,5).

Since efforts to promote adoption of guidelines through multimodal educational interventions alone may not result in improved adherence to standard of care (6), additional measures may be warranted to ensure adherence to the Toronto Consensus Statements. Computerized physician order entry systems can reduce the incidence of preventable adverse events and medication errors and optimize patient safety and efficiency outcomes (7,8). At our institution, order sets have previously been used in the emergency room to streamline medical care and avoid medical errors. The use of standardized protocols permits easier teaching of residents, adoption of approaches by staff, as well as auditing (9). In multiple studies, they have been shown to increase the adherence to standards of care including ordering of appropriate tests, improving consistency of management, and increasing prompt management (10,11). However, there are limited data on their use of patients hospitalized with IBD. We developed a quality improvement initiative which involved the implementation of a standardized order set for patients hospitalized with a severe UC flare and conducted a retrospective study assessing adherence to guidelines before and after the intervention. We hypothesized that implementation of the standardized admission order set could result in improved adherence to current best practice guidelines in the management of this patient population.

## METHODS

### Study Design and Setting

Adult patients presenting to the emergency department of an urban adult tertiary care university-affiliated teaching center in Montreal, Canada and admitted with a diagnosis of severe UC flare were identified for this retrospective cohort study. Two cohorts were established based on implementation of a standardized order set, which became available in March 2017. The pre-intervention cohort comprised of patients hospitalized between April 1, 2014 and October 31, 2016, and the post-intervention cohort participants were admitted between July 1, 2017, and June 30, 2018. The intervention consisted of a standardized patient order set accessible to the attending emergency medicine physician that could be used when admitting a patient with a severe UC flare to the short stay wards. These wards are run by family medicine physicians and emergency medicine physicians, as gastroenterology does not admit at our institution. A minority of patients were admitted to the internal medicine wards. The order set was developed by all co-authors in collaboration with the administrative team of the hospital. Sections included admission information, consults, diagnostic tests, diet and nutrition, activity level, vital signs, monitoring of bowel movements, laboratory tests, glucose management, intravenous therapy, antibiotic therapy, corticosteroid therapy, nausea management, pain/fever management, and VTE prophylaxis. Additional orders could be entered. Suggested orders were already selected upon opening of the order set, and included stool cultures, *C difficile* stool testing, methylprednisolone 20 mg intravenous every 8 hours for 72 hours, non-opioid pain control with acetaminophen, and weight-appropriate thromboprophylaxis using low-molecular-weight-heparin, with instructions that these should be considered for all patients admitted with a severe UC flare unless contraindicated. In addition, a gastroenterology consultation to assess the patient for sigmoidoscopy and co-management of the patient, and infectious diseases consultation for the evaluation of latent tuberculosis status were encouraged. The final version of the standardized order set can be found in the Supplementary Material.

### Eligibility Criteria

Charts of patients presenting with a principal diagnosis of ‘Colitis’, ‘Ulcerative Colitis’, ‘Acute Abdomen’, and ‘Peritonitis’ on the Canadian Emergency Department Diagnosis Shortlist were manually reviewed for inclusion by three investigators (12). Validation of an admitting diagnosis of severe UC flare required the patient to carry a diagnosis of UC prior to admission or to be suspected of having UC at presentation with the diagnosis being confirmed during the admission. Severe UC flare was defined as per the criteria put forth by Truelove and Witts (13).

### Study Variables

Information on baseline characteristics of the study population was collected from patient medical records via a retrospective chart review and included age, sex, known UC diagnosis, number of hospitalizations in the past year, hemodynamic instability at presentation, gastrointestinal bleeding, and biomarker levels (erythrocyte sedimentation rate , C-reactive protein [CRP], and hemoglobin). Quality indicators (QIs) evaluated for adherence to best practice guidelines included diagnostic testing, and both pharmacological and non-pharmacological interventions based on those described in the Toronto Consensus Statements (1). Laboratory, microbiology, and imaging investigations including *C difficile* toxin testing, stool culture, abdominal X-ray, flexible sigmoidoscopy within 72 hours of presentation, CRP within 24 hours of presentation, testing for latent tuberculosis (by interferon-gamma release assay or tuberculin skin test) were recorded. We also evaluated the administration of thromboprophylaxis within 24 hours of presentation, corticosteroids total daily dose-equivalent to 60 mg of methylprednisolone, and intravenous infliximab given after 5 days of corticosteroids at an appropriate dosage. Moreover, avoidance of narcotics and the request for a surgery consultation after 5 to 7 days of therapy in non-respondents were also recorded. The proportions of admissions with adherence to individual components of the quality indicators (QI) were measured.

### Outcome

Our primary outcome was defined as the difference in adherence to each of the 11 individual quality indicators in the pre-intervention group compared to the post-intervention group. Adherence was defined as the proportion of the quality indicator that was completed for each cohort.

### Sample Size Calculation

Thromboprophylaxis was selected as a proxy for the other quality indicators to be used to complete a sample size estimate. Described adherence rates to thromboprophylaxis during hospitalization for IBD flare are as low as 37% (14). Considering an adherence rate of 40% for our patient population, our sample size would need to be of 56 patients in total to demonstrate an increase to 80% adherence in the post-intervention cohort, which we thought would represent a clinically relevant improvement for what investigators felt was one of the most important quality indicators.

### Statistical Analyses

Individual hospitalization episodes were considered as the unit of analysis. Categorical data were expressed as proportions and continuous data as means ± standard deviations (SD). For comparison of adherence to quality indicators, between-group analysis was performed using Chi-square tests or Fisher exact tests for categorical variables; continuous variables were analyzed using *t*-tests or non-parametric Wilcoxon signed-rank tests as appropriate. A *P*-value < 0.05 was considered statistically significant. Missing data were not imputed. All statistical analyses were performed using SAS 9.4 (SAS Institute, Inc., Cary, NC, USA).

### Ethics

The institutional review board of the Jewish General Hospital gave approval for data collection and analysis.

## RESULTS

### Baseline Characteristics

An initial search of emergency department electronic medical records yielded 302 and 183 potentially eligible hospitalizations in the pre- and post-intervention cohorts, respectively. After detailed chart reviews, we included a total of 49 and 37 hospitalization episodes in the pre- and post-intervention cohorts, respectively. Excluded hospitalizations were those of patients who did not have a severe UC flare. Overall, participants in both groups were similar (Table 1). However, patients in the post-intervention group were more commonly women, had fewer prior hospitalizations, and had a higher CRP than those in the pre-intervention group (Table 1). The standardized order set was used in 24/37 of admissions in the post-intervention cohort (64.8%).

**Table 1.** Baseline characteristics of patient admissions

	Pre-intervention *N* = 49	Post-intervention *N* = 37
Age, mean ± SD	43.9 ± 18.2	39.5 ± 16.0
Female, *n* (%)	19 (38.8%)	21 (56.8%)
Known for UC, *n* (%)	46 (93.9%)	34 (91.9%)
Number of admissions per patient in past year, mean ± SD	2.7 ± 4.5	0.2 ± 0.5
Hemodynamic instability, *n* (%)	2 (4.1%)	3 (8.1%)
CRP at presentation (mg/L), mean ± SD	46.0 ± 61.0	87.2 ± 86.9
Hemoglobin (g/L), mean ± SD	107.5 ± 28.8	120.7 ± 23.8
Diarrhea, *n* (%)	46 (93.9%)	37 (100%)
Evidence of gastrointestinal bleeding, *n* (%)	44 (89.8%)	33 (89.2%)
More than six bowel movements, *n* (%)	38 (82.6%)	35 (100.0%)

CRP = C-reactive protein; SD = Standard deviation; UC = Ulcerative colitis.

### Adherence to Quality Indicators

Adherence to 6 of the 11 quality indicators was improved in the post-intervention cohort. Significant increases after implementation of the standardized order-set were noted in adherence to *C difficile* testing (75.5% versus 91.9%, *P* < 0.05), CRP testing (71.4% versus 94.6%, *P* < 0.01), testing for latent tuberculosis (38.1% versus 84.6%, *P* < 0.01), thromboprophylaxis (28.6% versus 94.6%, *P* < 0.01), adequate corticosteroids prescription (72.9% versus 94.6%, *P* < 0.01), and limitation of narcotics prescribed (68.8% versus 38.9%, *P* < 0.01). There were no significant differences in stool cultures performed, flexible sigmoidoscopy within 72 hours, abdominal X-ray, or use of infliximab as a rescue therapy. Appropriate surgical consultation after inadequate response to 5 to 7 days of medical therapy was identical in both groups (Table 2).

**Table 2.** Comparison of adherence to quality indicators in the pre- and post-intervention cohorts

Quality indicator	Adherence pre-intervention *N* = 49	Adherence post-intervention *N* = 37	*P*-value
*Clostridioides difficile* toxin testing	37 (75.5%)	34 (91.9%)	<0.05
Stool culture performed	37 (75.5%)	31 (83.8%)	0.35
Abdominal X-ray performed	19 (38.8%)	15 (42.9%)	0.71
Flexible sigmoidoscopy within 72 h of presentation	16 (32.7%)	13 (35.1%)	0.81
C-reactive protein performed within 24 h of presentation	35 (71.4%)	35 (94.6%)	<0.01
Testing for latent tuberculosis performed or previously documented	16 (38.1%)	22 (84.6%)	<0.01
Venous thromboembolism prophylaxis initiated within 24 h of presentation	14 (28.6%)	32 (86.5%)	<0.01
Intravenous steroids at a dose-equivalent of 60 mg of solumedrol in total	35 (72.9%)	35 (94.6%)	<0.01
Infliximab given after 5 days of corticosteroids, intravenous route, with proper dosage	5/15 (33.3%)	5/14 (35.7%)	1.00
Narcotics prescribed	33 (68.8%)	14 (38.9%)	<0.01
Surgery consultation after 5–7 days of therapy	3/3 (100.0%)	3/3 (100.0%)	1.00

## DISCUSSION

In this study, we assessed adherence to the Toronto Consensus Statement guidelines on the management of patients hospitalized with severe UC by examining the quality indicators achieved per patient admission after implementing a standardized order set and comparing the pre- and post-intervention cohorts. We demonstrated a clinically and statistically meaningful difference in hospital admissions to 6 of the 11 quality indicators in the post intervention cohort, including *C difficile* toxin testing, CRP measurement performed within 24 hours of presentation, testing for latent tuberculosis performed or previously documented, VTE prophylaxis initiated within 24 hours of presentation, intravenous steroids administration at a dose-equivalent of 60 mg of solumedrol in total, and avoidance of narcotics prescribing. Consistent with previous studies (15), VTE prophylaxis initiation within 24 hours of presentation showed the greatest improvement in adherence following the implementation of the order set. In a post-hoc analysis, the power was calculated to be 100% for this outcome assuming an alpha of 0.05. The power was lower for the other outcomes.

Previous studies have shown the benefits of implementing standardized templates in the care of patients with IBD. A health maintenance template in the electronic medical record system of an outpatient gastroenterology clinic serving the Veteran’s Affairs IBD population increased the recommendations for preventative care measures such as vaccinations, tuberculosis screening, osteoporosis screening, smoking cessation, and colorectal cancer surveillance by 22.5% to 86.3% (16). Similar improvements in adherence to influenza and pneumococcal immunization as well as tobacco cessation in the outpatient IBD population have been described when incorporating an IBD-specific note template, an order set, and a patient education intervention without any subsequent decrease in documented adherence a year following the intervention (17). Moreover, physician education in addition to a template summarizing IBD quality measures increased adherence to quality measures in both the academic and private practice outpatient settings (18). While the impact of standardized order sets for patients with IBD in the outpatient settings has been well described, the use of standardized order sets for hospitalized patients remains scarce. In one study, an order set for the management of patients presenting with acute severe ulcerative colitis in the ED setting significantly reduced time to *C difficile* ordering and time to infliximab administration (19). Similarly, an inpatient protocol for patients admitted with UC comprising order sets for gastroenterology consultation, daily gastroenterology progress notes, and standard recommended diagnostic tests and treatments, in addition to an opiate analgesic education and awareness campaign effectively increased the composite outcome of adherence to *C difficile* testing, thromboprophylaxis initiation, and opiates avoidance (20). Our study is in keeping with these previous findings and demonstrates improved adherence to six individual quality markers with respect to diagnostic tests and management in the hospital setting.

Whether the use of templates and data sets can lead to improved clinical outcomes remains understudied. One quality improvement strategy demonstrated that increased *C difficile* testing in patients with IBD was associated with shorter hospital stays and fewer admissions (21). In addition, the use of structured algorithms in the management of patients with IBD reduced ED visits (22). While it can be inferred that increased adherence to best practices guidelines is likely to improve clinical outcomes, future studies will need to be conducted to assess the effects of standardized order sets on clinical outcomes in IBD including hospitalization duration, venous thromboembolic disease, and surgical complications. We did not observe significant differences in appropriate infliximab use after a course of ineffective appropriate therapy. Indeed, accurate use of infliximab was only used in 33.3% of scenarios in both cohorts, likely driven by delayed initiation. This suggests that there may be systemic issues beyond ordering that need to be addressed.

There are several strengths to our study. We report improvement in 6 out of 11 individual quality indicators with an easy-to-use order set that was mostly used by emergency and primary care physicians. This improvement is observed with only 64.8% of post-intervention admissions using the standardized order set. Our study was limited by the proportion of adherence to the intervention, likely due to the new introduction of such order sets and admitting teams not being accustomed to identifying and completing them. Given incomplete adherence to the order set, causality of intervention and improved adherence could not be robustly ascertained. We hypothesize that some of the reasons for not using the order set may have included lack of awareness of the order set, challenge in changing practice, and difficulty accessing the order set (23). Further study would be required to explore why the order set was not used for some cases at our institution, in order to improve its implementation into clinical practice. Moreover, despite our best efforts to alert and inform admitting physician, there was no reminder system or trigger to prompt them to use the order set. Thus, adherence score may be improved with additional interventions including physician education and increased familiarity with the order set. While we cast a broad net by screening participants using all diagnoses of emergency department presentation that may have applied, including ‘colitis’, ‘ulcerative colitis’, ‘Acute abdomen’, or ‘peritonitis’, patients who did not carry one of the pre-defined principal diagnoses may have been misclassified as not having a severe UC flare. However, misclassification of cases with severe UC flare were unlikely to have differentially affected the pre- and post-intervention cohorts, and as such this possibility is unlikely to have affected the internal validity of our study. The pre- and post-intervention analysis inherently may not account for changes in clinical practice independent of our intervention or for any possible Hawthorne effect. Furthermore, as the pre- and post-intervention cohorts were not randomized due to the study design, there were slight differences in baseline characteristics, possibly due to low sample size. There were 2.7 ± 4.5 admissions per patient in the past year in the pre-intervention cohort compared to 0.2 ± 0.5 admissions in the post-intervention cohort. Each hospitalization was considered independently to best evaluate the adherence of clinicians for each admission however some quality indicator orders may not be pertinent for recent repeat admissions, thereby lowering the reported adherence to the individual quality indicator. Indeed, while disease severity was not formally assessed, the higher CRP at presentation and greater number of patients with >6 stools per day suggest a possibly more severe group in the post-intervention cohort. Our study was limited to 1 year of use of the order set. Further studies would be warranted to evaluate whether improvement was sustained, and to evaluate long term outcomes following use of the order set. Given the single-center study design, additional studies would be required at other centers to assess generalizability of our findings. Finally, it is important to consider that treating the Toronto Consensus Statements as quality indicators ignores the intricacies of the Grade approach to creating guidelines and is considered a limitation of the study. However, all 11 statements examined were based on graded levels of evidence that yielded a strong recommendation by the experts and should therefore be considered as valuable proxies for quality indicators (1).

## Conclusions

Implementation of a standardized order set, focused on specific pre-defined quality indicators for hospitalized patients with severe UC, was associated with meaningful improvements to most quality indicators defined by the Toronto Consensus Statements. Further studies are warranted addressing similar admission order sets that target the management of patients admitted for other gastrointestinal conditions.

## Supplementary Material

gwac032_suppl_Supplementary_Checklist

## Data Availability

The data underlying this article will be shared on request to the corresponding author.
