# Supplementary material for: A Simple Admission Order-set Improves Adherence to Canadian Guidelines for Hospitalized Patients With Severe Ulcerative Colitis
Source: J Can Assoc Gastroenterol. 2023 Feb 4;6(3):131–5. doi: 10.1093/jcag/gwac032 (PMC10235590; doi:10.1093/jcag/gwac032)
Supplement: gwac032_suppl_Supplementary_Checklist [file gwac032_suppl_supplementary_checklist.pdf]

Hôpital général juif
Jewish General Hospital

Weight: Height:

Allergies:

Severe Ulcerative Colitis Admission Order Set

INITIALS

Admission

Admit to: ______________________ Dr. ____

Diagnosis: **Severe Ulcerative Colitis Flare**

Comorbidities: 1. ____
2. ____
3. ____
4. Other: ____

Estimated Length of Stay: ☐ Less than 1 week ☐ Less than 10 days ☐ Less than 2 weeks

☒ Level of Intervention (LOI): _________ (Type LOI Level from list below) ☐ Not discussed (assumed Level 1)

☐ 1: Maximal interventions ☐ LOI order sheet completed

☐ 2A: Maximal interventions with restrictions, CODE BLUE called, CPR performed

☐ 2B: Maximal interventions with restrictions, CODE BLUE called, **NO** CPR

☐ 3: **NO** code called, maximal interventions to treat reversible conditions only, **NO** CPR, **NO** transfer to Critical Care Unit

☐ 4: **NO** code called, comfort care only, **NO** CPR, **NO** transfer to Critical Care Unit

Restrictions: ____

Discussed with: ________________________________ Date: _______________ Time: _______________

Home Medication

☐ No current medication

☐ Medication:

1. ____ ☐ Continue ☐ Hold
2. ____ ☐ Continue ☐ Hold
3. ____ ☐ Continue ☐ Hold
4. ____ ☐ Continue ☐ Hold
5. ____ ☐ Continue ☐ Hold
6. ____ ☐ Continue ☐ Hold
7. ____ ☐ Continue ☐ Hold
8. ____ ☐ Continue ☐ Hold
9. ____ ☐ Continue ☐ Hold
10. ____ ☐ Continue ☐ Hold
11. ____ ☐ Continue ☐ Hold

Completed by: ____________________ ________ ____________ ______________________
Name, Designation License # Signature Date Time

Verbal Order: ____________________ ________ ____________ ______________ ☐ Read Back
Ordering Practitioner's Name, Designation License # Signature Date Time

Transcribed by: ____________________ ________ ____________ ______________________
Name, Designation License # Signature Date Time

Verified by: ____________________ ________ ____________ ______________________
Name, Designation License # Signature Date Time

Hôpital général juif
Jewish General Hospital

Weight: Height:

Allergies:

Severe Ulcerative Colitis Admission Order Set

INITIALS

Consults

MD to complete paper request form

☒ GI – Reason: Severe UC follow care and **possibility of flexible sigmoidoscopy**

☒ Allergy – Reason: Assess latent TB status with PPD

☐ Infectious Disease – Reason: ____________________

☒ Dietitian – Reason: **Severe UC, assess for malnutrition**

☐ Surgery – Reason: ____________________

☐ Physiotherapy – Reason: ____________________

☐ Social Worker – Reason: ____________________

☐ Discharge Planning – Reason: ____________________

☐ ____________________ – Reason: ____________________

Diet and Nutrition

*** If NPO assess need for dietician or TPN consult***

☒ Low residue and lactose free ☐ NPO only if agreed by GI team

General Diet options

☐ Regular ☐ Diabetic ☐ Cardiac ☐ Renal ☐ Other: ____________________

Activity

☒ AAT

☐ Other: ____________________

Vitals

☒ Weigh q week

☒ T°, HR, RR, BP, SpO_2, Pain Score q shift and PRN

☐ T°, HR, RR, BP, SpO_2, Pain Score q4h and PRN

☒ If T° ≥ 38.5°C PO, notify MD

- ☒ Blood culture x 2,
- ☒ Urine analysis
- ☒ Urine culture

Maximum once per 24 hour period if continuing to spike T° ≥ 38.5°C

- ☒ Chest x-ray to be done (MD to complete requisition)

☐ Other: ____________________

Completed by: ____________________ ____________ ____________ ____________
Name, Designation | License # | Signature | Date | Time

Verbal Order: ____________________ ____________ ____________ ____________ ☐ Read Back
Ordering Practitioner's Name, Designation | License # | Signature | Date | Time

Transcribed by: ____________________ ____________ ____________ ____________
Name, Designation | License # | Signature | Date | Time

Verified by: ____________________ ____________ ____________ ____________
Name, Designation | License # | Signature | Date | Time

Hôpital général juif
Jewish General Hospital

Weight: Height:

Allergies:

Severe Ulcerative Colitis Admission Order Set

INITIALS

Respiratory

Oxygen Therapy

☒ Titrate O_2 to achieve a target SpO_2 > 90% **OR** ☐ Titrate O_2 to achieve a target SpO_2 ______ to ______ %
O_2 flow by nasal cannula (preferable, max 5 Lpm) or by Venturi/Venti/High Humidity mask (max 50%)
☒ Actively reduce O_2 flow/FiO_2 to minimal amount needed to meet SpO_2 target
- Refer to O_2 therapy table

☒ Notify MD if
- ☒ FiO_2 > 50% (or 6 Lpm via nasal cannula)
- ☒ Patient demonstrates respiratory fatigue or decreased level of consciousness

Patient with known chronically elevated PaCO2
☐ Titrate O_2 to achieve a target SpO_2 88 - 92% **OR** ☐ Titrate O_2 to achieve a target SpO_2 ________ to ___________%
Patient with known OSA
☐ Patient to use home CPAP
- ☒ Refer to Respiratory Nocturnal PAP pre-printed orders *****MD to complete*****

☐ Other: ____

Monitoring Bowel Movements

☒ Document
- ☒ Number of bowel movements (BM)
  - Day
  - Night
- ☒ Consistency of BM
  - Formed
  - Semi formed
  - Liquid
- ☒ Proportion of BMs with blood
  - Few BMs have blood
  - Less than half of BMs have blood
  - Most BMs have blood

Completed by: ____________________ ________ ____________ ____________________
Name, Designation | License # | Signature | Date | Time

Verbal Order: ____________________ ________ ____________ ____________ ☐ Read Back
Ordering Practitioner's Name, Designation | License # | Signature | Date | Time

Transcribed by: ____________________ ________ ____________ ____________________
Name, Designation | License # | Signature | Date | Time

Verified by: ____________________ ________ ____________ ____________________
Name, Designation | License # | Signature | Date | Time

Hôpital général juif
Jewish General Hospital

Weight: Height:

Allergies:

Severe Ulcerative Colitis Admission Order Set

INITIALS

Laboratory

Chemistry (light green top tube)

☒ CHEM 10

- ☒ NA ☒ K ☒ CL ☒ BIC
- ☒ UREA ☒ CRE ☒ GLUR ☒ CA
- ☒ MG ☒ PHO

☒ LFT

- ☒ ALK Alk. Phos
- ☒ ALT ALT
- ☒ BILT Bilirubin total

☒ GGT GGT

☒ AMYP Amylase (pancreatic)

☒ CRP C Reactive Protein

Hematology (lavender tube)

☒ CBC + auto-diff

☐ Other: ______________________________

☒ **REPEAT CHEM10, CRP and CBC: daily for 3 days then twice a week**

Completed by: ________________ Name, Designation | ________ License # | ________ Signature | ________ Date Time

Verbal Order: ________________ Ordering Practitioner's Name, Designation | ________ License # | ________ Signature | ________ Date Time ☐ Read Back

Transcribed by: ________________ Name, Designation | ________ License # | ________ Signature | ________ Date Time

Verified by: ________________ Name, Designation | ________ License # | ________ Signature | ________ Date Time

Hôpital général juif
Jewish General Hospital

Weight: Height:

Allergies:

Severe Ulcerative Colitis Admission Order Set

INITIALS

Laboratory Continued…

Microbiology

Send with microbiology blue requisition

☒ Stool culture
☒ C. difficile stool sample

Diagnostics

Please ensure requisitions are completed and faxed

☒ CXR PA + Lateral Reason: R/O latent TB ______________________________
☒ Abdominal series (AXR + CXR) Reason: R/O dilated colon ______________________________
☐ CT Abdo Pelvis (eg. Rule out abscess or perforation) Reason: ______________________________
☐ ________________________ Reason: ______________________________

IV Therapy

Bolus IV

☐ 0.9% NS ________ mL over ____________
☐ Other: ______________________________

IV Fluid

☐ 0.9% NS ☐ D5 and 1/2 NS ☐ Other: ________________________ at ________ mL/h
WITH ☐ 20 mmol KCl/L
WITH ☐ 40 mmol KCl/L
☒ Discontinue IV when drinking well. If on IV medications, then change IV to NS Lock

MRSA Prophylaxis

☒ Mupirocin 2% ointment apply Topically to nares BID
☒ Chlorhexidine 4% soap: wash body twice weekly
☒ If contraindication to Chlorhexidine 4%: Triclosan soap

Antibiotic Therapy

No antibiotic recommended. If prescribed reason mandatory
☐ ______________________________
☒ Reason: ______________________________

Completed by: ____________________ ____________ ____________ ____________
Name, Designation License # Signature Date Time

Verbal Order: ____________________ ____________ ____________ ____________ ☐ Read Back
Ordering Practitioner's Name, Designation License # Signature Date Time

Transcribed by: ____________________ ____________ ____________ ____________
Name, Designation License # Signature Date Time

Verified by: ____________________ ____________ ____________ ____________
Name, Designation License # Signature Date Time

Hôpital général juif
Jewish General Hospital

Weight: Height:

Allergies:

Severe Ulcerative Colitis Admission Order Set

INITIALS

Corticosteroid Therapy

☒ Methylprednisolone Succinate (Solumedrol®) 20 mg IV q8h

*****After 3 days of treatment, reassess for clinical, biochemical, or radiological lack of response or deterioration. If so, consider, along with the GI team, rescue therapy with Infliximab (Remicade®) or surgery. *****

☐ Other: ______________________________

Glucose Management

☒ CBGM QID for 24 hours then re-assess

Nausea Management

*****MD to consider ordering low dose for the elderly/frail*****

☐ Dimenhydrinate (Gravol®) 50 mg PO//IV q6h PRN (max 400 mg/day)

Pain / Fever Management

Non-opioids

☐ Acetaminophen 1,000 mg PO q6h PRN **(max 4,000 mg in 24 hours)**

Opioids

Opioids should be avoided. If prescribed, reason mandatory.

☐ ______________________________

☒ Reason: ______________________________

Completed by: ____________ ____________ ____________ ____________
Name, Designation | License # | Signature | Date | Time

Verbal Order: ____________ ____________ ____________ ____________ ☐ Read Back
Ordering Practitioner's Name, Designation | License # | Signature | Date | Time

Transcribed by: ____________ ____________ ____________ ____________
Name, Designation | License # | Signature | Date | Time

Verified by: ____________ ____________ ____________ ____________
Name, Designation | License # | Signature | Date | Time

Hôpital général juif
Jewish General Hospital

Weight: Height:

Allergies:

Severe Ulcerative Colitis Admission Order Set

INITIALS

VTE Prophylaxis

*****Universal VTE prophylaxis is standard of care in this patient population and should be universally prescribed, unless hemorrhagic shock or absolute contraindication. Document reasons for no VTE prophylaxis. MD to reassess VTE Prophylaxis daily if not ordered*****

*****LMWH is preferred due to decreased risk of Heparin Induced Thrombocytopenia*****

*****If surgery is anticipated in the next 12 – 24 hours consult surgery or anesthesia*****

☐ No Pharmacological VTE Prophylaxis - Reason: ☐ Patient on therapeutic anticoagulation
☐ Other: ______________________________

Pharmacological Prophylaxis

☒ Enoxaparin 40 mg SC daily until discharge

For CrCl < 30 mL/minute

☐ Enoxaparin 30 mg SC daily until discharge

If weight ≥ 120 kg:

☐ Enoxaparin 30 mg SC bid until discharge

☐ Other: ______________________________

Additional Orders

____

____

____

____

____

____

____

____

Completed by: ______________ ______________ ______________ ______________
Name, Designation | License # | Signature | Date | Time

Verbal Order: ______________ ______________ ______________ ______________ ☐ Read Back
Ordering Practitioner's Name, Designation | License # | Signature | Date | Time

Transcribed by: ______________ ______________ ______________ ______________
Name, Designation | License # | Signature | Date | Time

Verified by: ______________ ______________ ______________ ______________
Name, Designation | License # | Signature | Date | Time
